# Exploring presenteeism trends: a comprehensive bibliometric and content analysis

**DOI:** 10.3389/fpsyg.2024.1352602

**Published:** 2024-05-20

**Authors:** Divyakala Chandrakumar, Vasumathi Arumugam, Asokan Vasudevan

**Affiliations:** ^1^VIT Business School, Vellore Institute of Technology (VIT), Vellore, India; ^2^INTI International University, Nilai, Negeri, Sembilan, Malaysia

**Keywords:** presenteeism, productivity, workplace, bibliometric review, psychological health, physical health

## Abstract

**Objective:**

This research will conduct a bibliometric and content analysis of presenteeism from 2000 to 2023. It aims to investigate publication trends, authorship patterns, and significant publications by using presenteeism conceptualizations, measurements, determinants, consequences, and interventions analysis. The study provides valuable insights for researchers, practitioners, and policymakers about understanding and addressing workplace presenteeism issues.

**Methods:**

The research involved conducting a bibliometric study to analyze presenteeism publication trends, authorship patterns, and significant publications. It also explored the evolution of presenteeism research over time, identifying contributing countries, institutions, and writers. The interdisciplinary nature of presenteeism research was emphasized, covering occupational health, psychology, management, and public health. The researchers have used VOS Viewer and R Studio (biblioshiny) for this study.

**Results:**

The study identified several elements influencing presenteeism, such as health issues, work-related factors, organizational culture, and individual characteristics. It further examined the impact of organizational policies, leadership support, employee assistance programs, and health promotion activities in reducing absenteeism and enhancing employee well-being. These findings highlight the importance of addressing these factors to mitigate presenteeism issues and promote a healthier work environment.

**Conclusion:**

This research identified deficiencies in presenteeism research and provided recommendations for future investigations in this field. It emphasized the need for standardized measures and methodologies, longitudinal studies to understand causality, and industry- and population-specific interventions. These insights can guide future research directions and interventions to address presenteeism issues in a rapidly changing work and research landscape.

## 1 Introduction

Presenteeism has become a big buzzword, and it addresses real concerns from the recent past that will seriously affect individual health and productivity. Presenteeism is gaining significant traction in academia and, more recently, in practitioner literature as a crucial component of the modern business environment. A quick development has been observed in academia by giving specific attention to the notion, particularly in the international environment. Presenteeism is a phenomenon in which people are physically present at work but are not completely engaged or productive, and it is gaining attention in organizational studies (Evans-Lacko and Knapp, 2016). It is a widespread problem with serious consequences for both people and organizations. It impacts decreased productivity, increases healthcare expenditures, and affects employee well-being (Haque, 2021). Understanding the elements contributing to presenteeism and its repercussions is critical for designing successful workplace methods to address these issues (Sander et al., 2023).

In this study, we intend to use bibliometric analysis and content analysis to investigate the body of literature on presenteeism issues in the workplace. We hope to obtain a better knowledge of the current state of research on presenteeism issues by studying publication trends, authorship patterns, and research themes. Furthermore, we intend to identify the important factors contributing to presenteeism and its effects, interventions, and methods taken to address this issue by a comprehensive review of relevant publications. We expect that by doing this study, we may add to the current body of evidence on workplace presenteeism issues and provide insights that will help improve the organizational policies, practices, and interventions, which will reduce presenteeism and boost employee well-being and productivity in the organization.

Researchers are increasingly concerned about presenteeism due to its potential consequences for the organization and its employees. Presenteeism is extensively defined as reporting to work while ill (Aronsson et al., 2000; Johns, 2010). Werapitiya and Opatha (2015) examined 40 articles to arrive at a wide-ranging meaning of presenteeism. Twenty-three out of forty articles reveal that presenteeism is present in the workplace despite the employees being sick. This evidence emphasized that most studies defined presenteeism as employees’ preference for the workplace despite feeling ill. Presenteeism is described as being physically present at the workplace despite having deficient physical or mental health problems (Wee et al., 2019). It is important for organizations and individuals (Burton et al., 2004). Presenteeism and absenteeism are workplace attendance behaviors (WABs) (Ruhle and Breitsohl, 2022). Presenteeism has recently received attention and is an important factor influencing organizational performance (Lohaus and Habermann, 2019). Compared to absenteeism, presenteeism decreases productivity and is deemed a much more expensive issue (Hemp, 2012). Maintaining employees’ physical and mental health has become essential for any organization, as employees are one of their most valuable resources (Ruhle et al., 2020). Depression is both pervasive and debilitating, and it is also related to increased absenteeism and presenteeism (Johnston et al., 2019). According to Dew et al. (2005), early identification of workplace stress is necessary because it contributes to work-related accidents. According to Johns (2010), presenteeism is not defined in the existing literature. The difficulty in defining and measuring the concept stems from its complexity. Although presenteeism is gaining popularity among HR management implementers and scholars, additional research is required to clarify the concept’s conceptualization (Johnston et al., 2019).

Health conditions are linked to lost productivity, and presenteeism is a significant element of the overall considerable cost of those environments. However, the lost productivity cost cannot be resolved at this point (Schultz et al., 2009). Presenteeism research has concentrated on its prevalence in various occupational groups, its determinants, and its effects on productivity. However, there are few studies on the consequences of presenteeism on health (Aronsson and Gustafsson, 2005). There is literature to sustain the view that presenteeism desires more attention (Dew et al., 2005). Presenteeism has been connected to various psychosocial outcome measures, including poor psychological fitness and employee welfare (Brown et al., 2011). Presenteeism harms team productivity as well as health, impacting significant financial costs. According to the literature, presenteeism could result in higher indirect labor costs and medical costs than absenteeism (Bockerman and Laukkanen, 2010).

The topic of presenteeism is now being explored and discussed, particularly in scholarly publications from Australia, the United States, the United Kingdom, and Europe (Werapitiya and Opatha, 2015). Table 1 presents a compilation of definitions of presenteeism provided by various authors over the years. Presenteeism is when individuals attend work while not performing optimally due to physical or mental health issues, personal distractions, or other circumstances (Johns, 2010; Soliman et al., 2017; Taylor et al., 2021). This definition serves as the foundation for understanding the multifaceted nature of presenteeism. Presenteeism, which has garnered increasing attention in organizational psychology (Ruhle et al., 2020) and public health discourse, refers to employees attending work despite being ill or experiencing other health issues. This phenomenon significantly affects workplace productivity, employee well-being, and organizational culture. The definitions range from simple descriptions of attending work while sick to more nuanced understandings involving reduced performance, goal-directed behavior, and the impact of health challenges on work effectiveness. These diverse perspectives highlight the evolving nature of presenteeism as a concept and its relevance in contemporary work environments.

**Table 1:** Definitions of presenteeism.

Sl.No	Authors	Year	Definitions
1	Johns G.	2010	“Presenteeism refers to attending work while ill.”
2	Hemp P.	2004	“Presenteeism—the issue of workers’ being on the task but, due to sick or other health problems, not completely effective—can change single productivity by one-third or more.”
3	Koopman et al.	2002	“Even when Employees exist at their jobs, they may understand reduced performance and less than usual quality of work- a notion known as lower presenteeism.”
4	Dew et al.	2005	“The spectacle of working through sick and injury.”
5	Aronsson et al.	2000	‘To designate the spectacle of people, despite grievances and sick health issues that should rapid rest and absenteeism from job, still spinning up at their jobs”
6	Aronsson and Gustafsson	2005	“Presenteeism is the phenomenon of employees who present at their work with ill health that requires not attending from work and rest.”
7	Cooper et al.	2018	“Consensus is evolving that presenteeism defines attending work when one is ill”
8	Karanika-Murray and Biron	2020	“Presenteeism as goal-directed and focused presence performance meant at easing transformation to work in the face of haggled health.”
9	Wee et al.	2019	“Presenteeism is considered by attending to work irrespective of lessened real or mental health problems.”
10	Ariza-Montes et al.	2021	“Presenteeism is described as attending work when sick and not performing work at full potential”
11	Yang et al.	2022	“Presenteeism is the practice of working while illness or undergoing emotional or cognitive difficulties.”
12	Ruhle, S. A. and Breitsohl, H.	2023	“Behavior of working in the state of ill-health”
13	Hung et al.,	2024	“Presenteeism is the phenomenon where a worker continues to attend work despite feeling unwell due to illness or fatigue caused by long working hours, leading to reduced productivity.”

### 1.1 Dimensions of presenteeism

The study by Werapitiya and Opatha (2015) identifies five key dimensions that characterize presenteeism: working while sick, exceeding required work hours, not fully engaging in assigned tasks, working on tasks unrelated to assigned work, and displaying overactive or hyperactive behavior in completing assignments. These dimensions encompass various behaviors and attitudes contributing to reduced productivity and potential negative outcomes for individuals and organizations. Understanding and addressing these dimensions are crucial for promoting healthier work environments and maximizing employee effectiveness. Analyzing each dimension through content analysis allows researchers to uncover underlying factors and trends. In contrast, bibliometric analysis tracks the evolution of research and highlights key studies or theories explaining presenteeism-related phenomena. By examining these dimensions, researchers can gain insights into the prevalence, causes, and potential interventions for presenteeism in different workplace contexts.

### 1.2 Scope of presenteeism

Presenteeism research has a broad scope that encompasses understanding the individual, interpersonal, and organizational factors influencing employees’ decisions to attend work while ill or experiencing health issues (Dietz et al., 2020). This research investigates the impact of presenteeism on various organizational outcomes, such as productivity, performance, job satisfaction, and turnover intentions. Bibliometric and content analysis are crucial in advancing presenteeism research by offering systematic and comprehensive insights into the existing literature and the conceptual landscape. Firstly, bibliometric analysis enables researchers to map the evolution of presenteeism literature over time, identifying key trends, seminal works, and emerging topics. By analyzing publication patterns, citation networks, and collaboration structures, researchers can have a better understanding of the field’s development, identify influential authors and journals, and pinpoint gaps or areas ripe for further investigation.

Additionally, content analysis was used by the researchers to delve into the thematic content of presenteeism literature, examining definitions, conceptual frameworks, measurement tools, and empirical findings in presenteeism studies. This approach facilitates the synthesis of diverse perspectives, identifying recurring themes or controversies, and validating theoretical constructs. By systematically analyzing the textual data, content analysis helps elucidate the complexities of presenteeism as a multifaceted phenomenon, offering valuable insights for theory development, empirical research design, and evidence-based practice in organizational and public health contexts. Bibliometric and content analysis provide methodological tools for comprehensively mapping the presenteeism research landscape, fostering knowledge accumulation, and guiding future research directions. This research domain explores interventions and organizational practices to promote a supportive work environment, encourage healthy behaviors, and effectively manage presenteeism to enhance employee well-being and effectiveness.

### 1.3 Research questions

**Figure 1:**
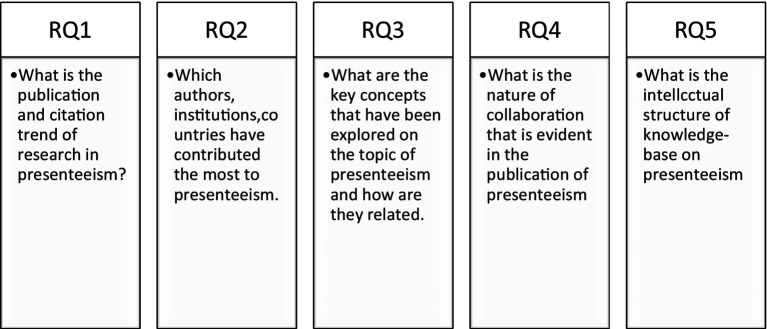
Research questions.

As noted by Ruhle et al. (2020), the limited literature support for presenteeism studies underscores the critical need for further research and reviews in this domain. As an emerging topic, presenteeism is increasingly recognized for its significant impact on organizational outcomes and employee well-being. While absenteeism has traditionally garnered more attention, presenteeism’s nuanced effects on productivity, performance, and health have become increasingly evident. However, despite its growing importance, there remains a gap in the literature regarding comprehensive reviews and theoretical frameworks that synthesize existing knowledge and identify key research questions. Figure 1 in this review article presents five major research questions based on a rigorous combination of bibliometric analysis and systematic literature review. The review aims to provide a comprehensive understanding of the motivations behind presenteeism and its positive and negative consequences, thus addressing a crucial gap in the presenteeism literature. By delving into the factors that drive employees to attend work while ill or experiencing health issues, as well as the outcomes associated with this behavior, the review seeks to contribute to a deeper understanding of presenteeism’s implications for both individuals and organizations.

## 2 Theoretical background

Presenteeism is defined as employees being physically present at work but not fully engaged or productive due to various factors, and it has garnered significant attention in organizational research (Patel et al., 2023). Conceptually, presenteeism encompasses the notion of reduced productivity or engagement while on the job despite the employees being physically present (Biron et al., 2022). Antecedents of presenteeism span individual, organizational, and contextual factors (Woodland et al., 2023). Individual factors such as health status and job satisfaction, organizational factors including workplace culture and leadership styles, and contextual factors like economic pressures and organizational policies play a role in contributing to presenteeism (Chen et al., 2024).

Research has extensively revealed the negative consequences of presenteeism at both individual and organizational levels. Individuals engaging in presenteeism are prone to experiencing decreased job satisfaction, burnout, and impaired health, ultimately leading to reduced performance (Lui et al., 2024). At the organizational level, presenteeism is associated with higher healthcare costs, decreased productivity, lower morale, and increased turnover rates. Consequently, understanding the antecedents and consequences of presenteeism is crucial for organizations to develop effective interventions and strategies to mitigate its impact (Ozduran et al., 2023).

Presenteeism, often overlooked in discussions about workplace productivity, can harm employees and organizations. When employees come to work while sick or experiencing personal issues, their ability to perform tasks effectively is compromised. This can lead to decreased productivity, increased errors, and lower quality of work. Moreover, working while unwell can prolong recovery time, potentially leading to more extended absences in the future and higher healthcare costs for both employees and employers (Mazzetti et al., 2019). Additionally, presenteeism can contribute to a negative work environment, as colleagues may become exposed to illnesses and feel pressure to work when resting.

To address presenteeism effectively, organizations need to implement policies and practices that prioritize employee well-being. This includes offering sufficient paid sick leave and encouraging employees to use it when necessary without fear of negative consequences. Flexible work arrangements, such as telecommuting options or adjusted work schedules, can help employees manage their workload while dealing with health issues or personal challenges (Bierla et al., 2013). Furthermore, fostering a culture that values work-life balance and supports open communication about health concerns can empower employees to take care of themselves and reduce the stigma associated with taking time off when needed.

Employers should also invest in proactive measures to prevent presenteeism, such as promoting healthy lifestyles through wellness programs and providing resources for managing stress and mental health (Karanika-Murray and Biron, 2020). By prioritizing their employees’ physical and emotional well-being, organizations can create a more resilient workforce and foster a positive work environment where employees feel valued and supported. Addressing presenteeism benefits individual employees and contributes to overall organizational success by enhancing productivity, reducing healthcare costs, and improving employee morale.

Addressing presenteeism typically involves multifaceted interventions targeting individuals, organizations, and broader societal factors (Itani et al. Uslukaya et al., 2022). Strategies may include promoting a healthy work-life balance, providing access to mental health resources, offering flexible work arrangements, and creating a supportive work environment where employees feel comfortable discussing health concerns (Kim et al., 2020). A concise review of the literature on presenteeism is given below.

### 2.1 Presenteeism vs. absenteeism

Absenteeism signifies the nonappearance of workers at work. However, presenteeism means that workers are present in the workplace. Still, their capability to achieve work is decreased due to real and psychological issues (Effects of Job Stressors, Stress Response, and Sleep Disturbance on Presenteeism in Office Workers) (Miraglia and Johns, 2016). Presenteeism is the “contrast of absenteeism” (Caverley et al., 2007; Collins and Cartwright, 2012). While illness absenteeism is related to absence from work for medical reasons, illness presenteeism relates to attending work during illness. Presenteeism has been described as having a better relationship with job-related issues than absenteeism (Matsushita et al., 2011).

### 2.2 Presenteeism and psychological factors

Psychological issues, such as emotional exhaustion and adverse strain, have been stated to be related to presenteeism (Miraglia and Johns, 2016). Emotional exhaustion can also affect long-lasting presenteeism (Baker-McClearn et al., 2010). Individuals who bang presenteeism frequently also consider their work atmosphere worrying and disappointing. However, in his study, he indicates that the adverse relationship of presenteeism with job performance is owing to the absence of adequate opportunity for health retrieval. Therefore, attending to a job during sickness or poor health conditions has adverse significances, such as decreased job performance and job engagement (Côté et al., 2021). Presenteeism is related to psychological suffering, reduced mental and physical health, and burnout (Quigley et al., 2022).

### 2.3 Presenteeism and professional relationships

Presenteeism has been linked to professional relationships, whether it is between employer and employee or between co-workers (Aronsson et al., 2000; Nielsen and Daniels, 2004; Prater and Smith, 2011; Nielsen and Daniels, 2016). Co-worker relationships can also influence presenteeism (Prater and Smith, 2011). Multiple studies indicated that employees with solid work relationships tend to have a sense of duty toward their colleagues (Lu et al., 2013). It is found that individuals will continue to go to work physically, even if they are mentally absent, because of the fear of job loss due to the economy, not being able to meet the financial obligations (financial stress), and since those employees want to maintain their professional relationships (Aronsson et al., 2000).

### 2.4 Presenteeism and productivity

In disparity to absenteeism, the view of presenteeism states performance loss from workers’ incapability to work at complete ability, and it is due to mental or real sickness, even though they are still working in the work circumstances (Rantanen and Tuominen, 2011). In modern years, the debate on workers’ performance has shifted from worker nonattendance to presenteeism (Zhou et al., 2016). Researchers contended that attending to be present at the workplace when sick leads to greater expense and adverse impact on employee performance than being absent (Hemp, 2012). In association with worker performance, presenteeism cuts worker performance and productivity (Zhou et al., 2016). Presenteeism has been considered due to its negative influence on work performance (Ferreira et al., 2019). Presenteeism has become a main occupational health issue as it signifies a less visible but important basis of productivity losses that can have better cost significance for workers and organizations (Quigley et al., 2022).

### 2.5 Presenteeism and performance

Job performance is the most significant and reviewed construct in industrial management and organizational behavior domains (Widera et al., 2010). It is described as a distinct behavior that workers perform, which is worthwhile for the organization and supports items (Ángeles López-Cabarcos et al., 2020). Performance is a record of outcomes caused by certain job functions or actions during a period (Krijgsheld et al., 2022). It is a multifaceted variable that can be managed from the viewpoint of in-role job satisfaction and pioneering job productivity(Henttonen et al., 2016). In-role job satisfaction signifies the work within the duties of employees (Widera et al., 2010). This requires an individual to display the proper behaviors to attain their productivity, which is aimed at the job role. State-of-the-art job performance directs activities beyond usual job demands to achieve novel results (Ali-Hassan et al., 2015). The performance of employees has become important due to the snowballing concern of human resources and organizational specialists concerning the level of results attained from workers (Hemakumara, 2020). Job performance is a variable that includes behaviors under employees that regulate the commitment to the organizational goals (Brborović et al., 2017).

### 2.6 Presenteeism and cost

Stewart et al. (2003) discussed that the expense of presenteeism is three times greater than absenteeism in the United States. The expense and performance loss from presenteeism has been higher than that from absence (Goetzel et al., 2004; Hemp, 2012). Several researchers have estimated the expenses associated with presenteeism, and some have recommended that these expenses exceed absence due to an illness. Research shows a prospective association between presenteeism and illness leave, demonstrating that attending work while being sick may be a risk issue for future absenteeism. Since presenteeism comprises adverse consequences for an individual employee and an employer, it is more important to define evidence for preventive procedures (Pichler et al., 2016).

### 2.7 Antecedents of presenteeism

The two areas of study are connected to profile-based and organizational issues. Among the significant individual predictors of presenteeism behavior, the studies reveal gender (Aronsson and Gustafsson, 2005), age, job performance (Caverley et al., 2007), and the type of engagement (Aronsson and Gustafsson, 2005) are recognized as significant factors of presenteeism leanings. Presenteeism, as mentioned in past research, is the opposite of absence. It is when a worker trusts their job, even though they are too ill, hassled, or abstracted to show their performance; the sensation of wanting to engage additional hours, even if the worker has no additional job to do (Nielsen and Daniels, 2004).

### 2.8 Theoretical advancement in presenteeism research

Theoretical advancements in presenteeism have evolved beyond traditional absenteeism-focused perspectives, with several frameworks offering deeper insights into its causes and consequences. Early research on presenteeism often conceptualized it as a unidimensional construct focused solely on attendance despite illness (Homrich et al., 2020). However, contemporary theories have adopted multidimensional frameworks that consider various factors influencing presenteeism (Kinman and Grant, 2021). These frameworks typically include individual, organizational, and contextual dimensions, allowing for a more comprehensive understanding of the phenomenon. Stigma theory has been applied to understand the social dynamics surrounding presenteeism, particularly concerning mental health conditions (Ruhle et al., 2020). Research in this area explores how stigma associated with certain health conditions may influence employees’ decisions to attend work while unwell, as well as the potential consequences of perceived stigma on workplace relationships and outcomes (Lohaus and Habermann, 2019). Theoretical advancements in presenteeism research recognize the importance of cultural and contextual factors in shaping attitudes and behaviors related to attendance despite illness. Cross-cultural studies have revealed variations in the prevalence and drivers of presenteeism across different national and organizational cultures, highlighting the need for context-specific approaches to understanding and addressing the phenomenon.

## 3 Methodology

Presenteeism-focused research mapping in the workplace was based on data extracted from the Scopus Database. The researchers considered Scopus the data source to ensure methodical reliability and inclusivity. A bibliometric review followed the guidelines (Massaro et al., 2016). A bibliometric review is an in-depth examination of published scientific literature in a field or research area. The statistical methods employed for the bibliometric analysis of presenteeism research encompass two primary procedures: Performance analysis and science mapping. Performance analysis focuses on publications’ volume and growth trajectory over time, including identifying prolific authors, affiliated institutions, countries, and sources. Science mapping utilizes bibliometric methods to determine the structure of a research area by grouping documents, authors, journals, and words. For science mapping, three types of analysis were conducted using VOS viewer software: co-occurrence network, bibliographic coupling, and co-citation network. Additionally, two types of analysis were performed using R-studio software: country collaboration map and thematic map. These bibliometric networks were constructed to visualize collaboration patterns and the conceptual structure of scientific research on presenteeism. The bibliographic coupling determines the relevance of research articles based on the frequency of shared-cited references. Bibliographic coupling works with the extracted research papers from the Scopus database, whereas co-citation analysis works with cited references. The researchers used content analysis in this study to gain a better understanding of concepts and recent findings. Content analysis is making “sense out of text data, divide it into text or image segments, label the segments with codes, examine codes for overlap and redundancy, and collapse these codes into broad themes.” Because bibliometric reviews alone do not grab the interest of young scholars due to their emphasis on citations, TCCM (Theory, Characteristics, Context, and Methodology) analysis can overcome this constraint (Sharma et al., 2020). As a result, this study also used the TCCM framework to extract the essence of recently published publications and incorporate the work of renowned scholars, in particular those who have more citations.

**Figure 2:**
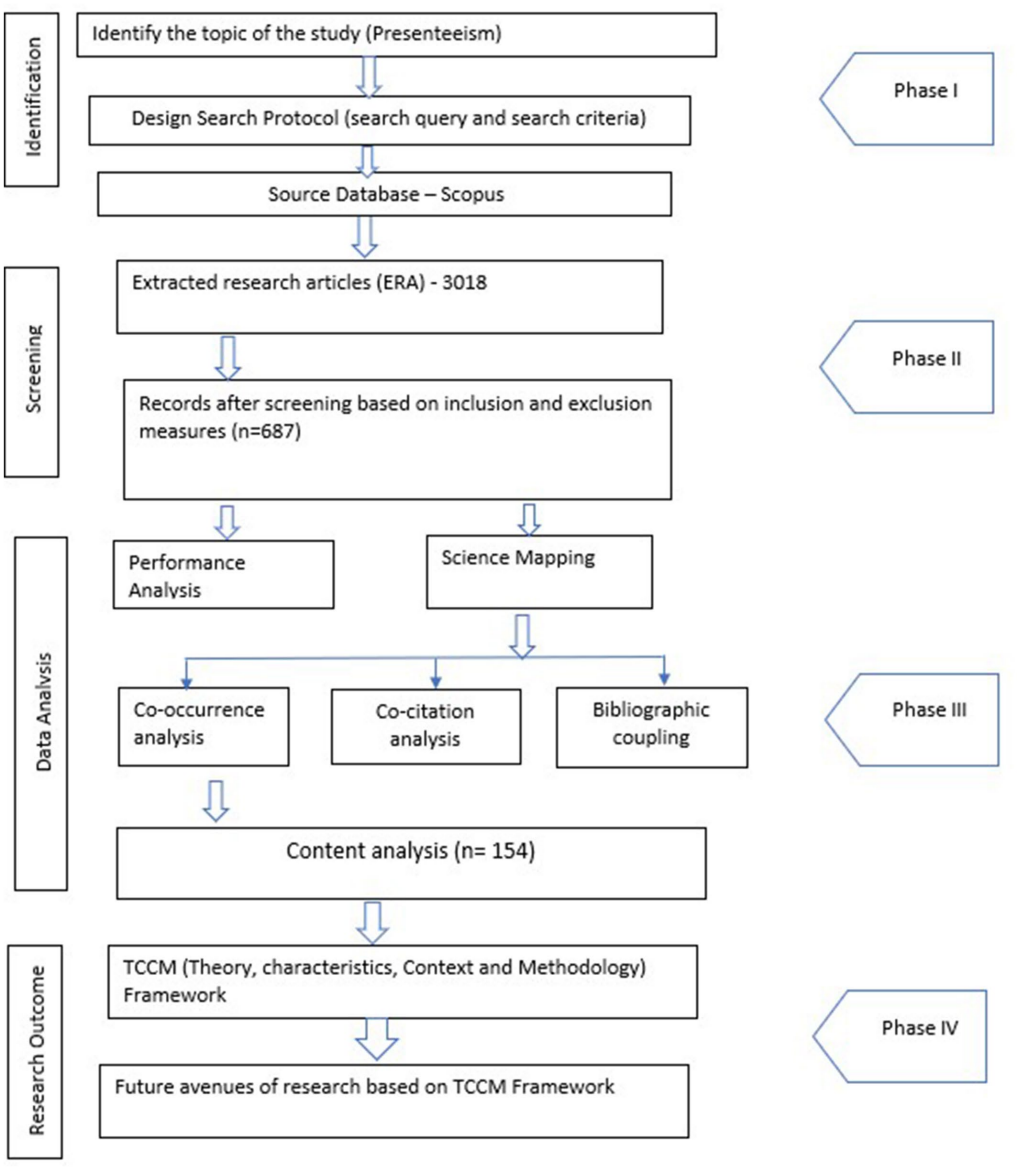
Methodology.

The study relied on a dataset of extracted research articles (ERA). The datasets are valuable for providing insights into presenteeism study. The co-citation analysis and bibliographic coupling were performed using the ERA dataset. The intellectual structure of presenteeism was retrieved in terms of several themes using content analysis of selected major research publications, co-citation analysis, and bibliographic coupling. The ERA dataset provides a comprehensive perspective because it is significant, and none of the articles can be overlooked. The selection of articles from each cluster of bibliographic coupling map and co-citation map reveals the fundamental characteristics of each cluster. Thus, 154 articles were chosen, and duplication was eliminated. It resulted in 154 significant research publications for content analysis. These publications are the most influential and important in the presenteeism study. The full process is depicted in Figure 2.

Content analysis was performed on abstracts from 154 recognized relevant research publications to determine the intellectual structure. The abstract was selected as the measure of analysis for content analysis because it represents the overall direction, reflection, and comprehension of the full research study. Several coding steps were used to conduct content analysis. In the first step, abstracts of designated key research publications were carefully examined, and codes based on meaningful words were assigned to abstract assertions. Then, after assigning codes to all assertions in all abstracts, all codes were assessed for their relationship with one another and categorized into themes. To ensure the accuracy of the findings regarding checking and external audit, both authors conducted a content analysis individually. Then, they checked each other’s findings before discussing and combining them into significant themes. After examining major research articles, it was discovered that the most current publications were from 2023. As a result, articles published after 2023 and classified as A*, A, and B journals according to the ABDC list 2022 were discovered, and the TCCM framework was applied to these articles, with 154 key research articles retrieved to capture a comprehensive knowledge of presenteeism research. The ABDC list was chosen since it is the most widely utilized and reputed journal quality list for literature review research published in various prominent journals. It comprises high-quality, top-ranked academic journals that are updated regularly, and premier business schools in many countries widely utilize it; thus, selecting this list contributes to the quality of the literature study.

### 3.1 Inclusion/ exclusion criteria

The bibliometric review has been undertaken to foster a better understanding of presenteeism in the workplace. Bibliometric analysis is also a robust technique for examining the evolution of study domains, which is an integral part of assessing academic production over many decades. In this study, the literature search process was employed by researchers using a systematic approach and various search criteria to retrieve relevant articles on presenteeism comprehensively. The researchers retrieved a total of 3,018 articles from the Scopus database by using title, abstract, and keyword searches focusing on terms such as “Presenteeism,” “working while ill,” “workplace presenteeism,” and “attendance culture.” These articles spanned various subject areas, including Business, Management, Accounting, Social Sciences, Psychology, Nursing, and Health Professions, resulting in 847 relevant articles. The researchers have narrowed the search to include only articles and reviews, yielding 752 documents.

Moreover, to focus specifically on scholarly journals, the researchers refined the search to include only articles published in journals, resulting in 748 articles. Further refinement ensured that only English-language publications were considered by the researchers, resulting in a final selection of 687 articles meeting the inclusion criteria. The researchers carried this rigorous search strategy to ensure a comprehensive collection of literature relevant to presenteeism, facilitating a robust analysis and synthesis of existing knowledge in the field.

## 4 Results

### 4.1 Bibliometric review

#### 4.1.1 Performance analysis

*RQ1:* What is the publication and citation trend of research in presenteeism?

Performance analysis in bibliometrics can assist academics, institutions, and funding agencies make informed decisions. Researchers can assess their performance, identify areas for improvement, and demonstrate their effect to future collaborators or employers. Institutions can examine their research production and impact, compare it to peers, and discover strengths and deficiencies. Funding organizations can utilize performance analysis to assess the impact of their investments, identify successful programs, and distribute resources efficiently. However, it is significant to highlight that bibliometric indicators have limits and should be used with qualitative assessments to evaluate research performance thoroughly (Table 2).

**Table 2:** Inclusion and exclusion measures.

S.No	Search criteria	No. of articles
1	TITLE-ABS-KEY (“Presenteeism” or “working while ill” or “workplace presenteeism” or “attendance culture”)	3,018
2	Subject area (business, management, and accounting; social sciences; psychology; nursing; health professions)	847
3	Document type (article; review)	752
4	Source type (Journal)	748
5	Publication stage (Final)	730
6	Language (English)	687

##### 4.1.1.1 Annual publication and citation

Table 3 represents the annual publication on the research area of presenteeism. Presenteeism is an emerging topic that has got the attention of researchers in recent years. The table implies that there is a growth of publications in this area. Presenteeism is one of the important aspects that must be considered in terms of organizational performance, productivity, and individual health. Until 2000, there was very little production of presenteeism in the workplace. The table below represents the details of the total output, Total citations per year, and cumulative total citations till the year 2023 were 11,498.

**Table 3:** Publication trends.

Year	TP	CTP	TC	CTC
2004	1	1	66	66
2005	3	4	415	481
2006	3	7	108	589
2007	3	10	196	785
2008	7	17	504	1,289
2009	10	27	795	2084
2010	7	34	948	3,032
2011	12	46	1,027	4,679
2012	11	57	431	5,110
2013	13	70	795	5,905
2014	16	86	439	6,344
2015	20	106	563	6,907
2016	25	131	970	7,877
2017	33	164	560	8,437
2018	32	196	1,012	9,449
2019	30	226	872	10,321
2020	32	258	770	11,091
2021	50	308	312	11,403
2022	47	355	75	11,478
2023	12	367	20	11,498

TP, total publication.

CTP, cumulative total publication.

TC, total citation.

CTC, cumulative total citation.

##### 4.1.1.2 Prolific authors, affiliated institutions, countries, and sources

*RQ2:* Which authors, institutions, countries, and sources have contributed the most to presenteeism.

The statement refers to Tables 4–6, which give detailed information on the most prolific authors, institutions, and nations in terms of citations in the field of presenteeism study. Presenteeism refers to the employees who are physically present at work but are not completely engaged or productive due to illness, stress, or personal difficulties. These tables contain information about the intellectual contributions and influence of researchers, institutions, and countries for a well-known understanding of presenteeism.

**Table 4:** Top 25 contributing authors in the field.

S.No	Author	TP	TC	TC/TP	Total link strength
1	Johns, G.	5	1,183	237	207
2	Schaufeli, W. B.	3	467	156	79
3	Ferreira, A. I.	10	369	37	169
4	Miraglia, M.	4	320	80	109
5	Martinez, L. F.	7	302	43	149
6	Cooper, C. L.	6	292	49	138
7	Watts, J. H.	3	278	93	0
8	Lu, L.	6	253	42	125
9	Zhang, W.	3	241	80	8
10	Lohaus, D.	5	209	42	156
11	Biron, C.	5	185	37	97
12	Karanika-Murray, M.	5	185	37	97
13	Sanderson, K.	3	175	58	14
14	Habermann, W.	4	144	36	141
15	Dietz, C.	4	122	31	45
16	Ruhle, S. A.	3	106	35	60
17	Caputi, P.	5	97	19	42
18	Yang, T.	7	89	13	59
19	Vinberg, S.	3	85	28	52
20	Correia Leal, C.	3	79	26	31
21	Zacher, H.	3	72	24	23
22	Li, Y.	4	59	15	59
23	Gillet, N.	3	52	17	8
24	Guo, S.	3	50	17	59
25	Wang, S.	3	50	17	59

TP, total publication.

TC, total citation.

TC/TP, cites per publication.

**Table 5:** Top 25 countries contributing to the field of presenteeism.

S.NO	Country	TP	TC	TC\TP	Total link strength
1	United Kingdom	82	3,067	37	739
2	United States	76	2,717	36	269
3	Canada	31	2,151	69	664
4	Australia	33	977	30	252
5	Netherlands	16	740	46	162
6	Germany	33	625	19	350
7	Denmark	8	528	66	155
8	Portugal	13	424	33	219
9	China	18	354	20	211
10	Taiwan	8	275	34	199
11	New Zealand	5	271	54	105
12	Sweden	12	190	16	107
13	Japan	18	177	10	29
14	Italy	9	154	17	76
15	Belgium	10	150	15	99
16	France	8	149	19	50
17	Hong Kong	4	139	35	27
18	South Korea	11	117	11	72
19	Spain	10	111	11	140
20	Singapore	5	87	17	2
21	Austria	5	79	16	48
22	Norway	10	77	8	63
23	Pakistan	5	73	15	38
24	Turkey	6	71	12	41
25	Switzerland	4	69	17	47

TP, total publication.

TC, total citation.

TC/TP, cites per publication.

**Table 6:** Top 25 Institutions contributing to the field.

S.NO	Institution	TP	TC	TC/TP	Total link strength
1	University of East Anglia, Norwich Business School.	2	238	119	38
2	Department of Business Administration, National Taiwan University, Taipei City, Taiwan	2	157	79	33
3	Lancaster University Management School, Lancaster University, Lancaster, United Kingdom	3	157	52	39
4	Nottingham Trent University, United Kingdom	3	117	39	23
5	Laval University, Canada	2	111	56	21
6	Nottingham Business School, Nottingham Trent University, Nottingham, United Kingdom	2	101	51	23
7	Manchester Business School, University of Manchester, United Kingdom	2	76	38	8
8	Department of Psychology, Nottingham Trent University, Nottingham, United Kingdom	2	65	33	20
9	Department of Business Administration, National Taiwan University, Taiwan	2	63	32	14
10	Lancaster University Management School, Lancaster, United Kingdom	3	61	20	14
11	Department of Psychology, Stockholm University, Stockholm, Sweden	2	50	25	8
12	Norwich Business School, University of East Anglia, Norwich, United Kingdom	2	46	23	2
13	Department of Clinical Neuroscience, Karolinska Institutet, Stockholm, Sweden	2	45	23	6
14	Nova School of Business And Economics, Universidad nova de lisboa, lisboa, Portugal	2	45	23	12
15	Centre for Organizational Health and Wellbeing, Lancaster University, Lancaster, United Kingdom	2	42	21	12
16	School for Professional Studies, Saint Louis University, United States	2	38	19	7
17	School of Public Administration, University of Victoria, Canada	2	38	19	4
18	University of Edinburgh Business School, the University of Edinburgh, Edinburgh, United Kingdom	2	34	17	4
19	College of Hospitality and Tourism Management, Sejong University, 98 Gunja-dong, Gwanjin-gu, Seoul, 143–747, South Korea	2	33	17	14
20	Faculty of Economics and Business, University of Barcelona, Spain	2	30	15	20
21	Department of Psychology, Norwegian University of Science and Technology (NTNU), Trondheim, Norway	2	28	14	0
22	Department of Psychiatry, Tokyo Medical University, Shinjuku-ku, Tokyo, 160–0023, Japan	2	23	12	0
23	Faculty of Social Sciences, University of Wollongong, Wollongong, Australia	2	23	12	4
24	School of Management, operations and Marketing, University of Wollongong, Wollongong, Australia	2	23	12	4
25	Institute of Psychology and Behavior, Henan University, Kaifeng, China	2	22	11	15

TP, total publication, TC, total citation, TC/TP, cites per publication.

Table 4 most likely lists the authors who contributed majorly to the presenteeism study. It could include their names, affiliations, the number of publications or articles they have written on presenteeism, and the citations those publications have earned. This table provides insights into the persons actively studying and contributing to the knowledge base concerning presenteeism by identifying the most prolific authors. Table 4 presents the authors who have received the highest number of citations in the field of presenteeism. According to the data, the most cited author is Johns (2010), with a total link strength of 207 and a citation count of 1,183. The next most cited author is Schaufeli et al. (2009), with 467 citations and a total link strength of 79. Ferreira et al. (2019) ranks third on the list with 369 citations and a total link strength of 169.

Table 5 presents the most cited countries in the field of presenteeism research. The United Kingdom leads with 3,067 citations and a link strength 739, indicating significant recognition and influence in the field. The United States follows closely with 2,717 citations, reflecting its substantial contributions to presenteeism research. Canada ranks third with 2,151 citations and a link strength of 664, highlighting its notable research impact. These three countries demonstrate their prominence and active involvement in advancing knowledge on presenteeism. The high citation counts suggest that other scholars have widely acknowledged and referenced research from these countries. The link strength values indicate the interconnectedness of studies from these countries with other presenteeism research. The data showcases the global distribution of scholarly work and the countries significantly contributing to a well-known understanding of presenteeism. Further analysis was carried out in Table 5, as given below, in a comprehensive view of the citation performance of other countries in this field.

Low-income countries like Bangladesh and Ghana stand out with singular contributions, yet Bangladesh’s four citations suggest its research has garnered attention despite limited output. Pakistan exhibits a more substantial involvement with six documents and 108 citations, indicative of a growing scholarly activity. Nigeria, with three contributions and citations, and Peru, with three papers and 29 citations, also demonstrate moderate engagement in the field. Other Low-income countries like Romania, Kazakhstan, Tunisia, and Uzbekistan each show a budding interest, with one or fewer documented contributions. While their impact may be limited, their presence suggests an emerging focus on presenteeism within their academic communities. These low-income countries are gradually becoming more involved in presenteeism research, though to varying extents. While some nations like Pakistan display a more robust scholarly output, others are still in the early stages of exploration. Nevertheless, their participation underscores a global recognition of the importance of understanding and addressing workplace health issues, even in resource-constrained environments. As these countries continue to contribute to the discourse, their perspectives and findings will enrich the broader understanding of the impact of presenteeism and potential interventions.

Table 6 reveals the institutions that have played a significant role in presenteeism research. It could include universities, research organizations, or other academic institutions that have conducted important studies on this domain. The table may include information such as the name and location of the university, as well as the number of publications or citations created by scholars affiliated with that institution. This chart identifies academic centres with devoted resources and expertise to enhance presenteeism research by identifying the main institutions. The University of East Anglia holds the first position with 238 citations, indicating that research articles affiliated with this institution have been highly influential and cited by other researchers in the field. The University of East Anglia has demonstrated a strong impact and a significant presence in advancing the understanding of presenteeism. In second place, the National Taiwan University is recorded with 157 citations. This indicates that the research articles affiliated with this institution have also substantially impacted and have been widely cited in presenteeism.

Frontiers in Psychology is a significant contributor, with 28 documents and 357 citations. It is crucial in advancing our understanding of presenteeism and its implications for individual well-being and organizational performance. The Journal of Occupational Health Psychology is another standout contributor, with 11 documents and 963 citations. Other notable contributors include the Journal of Occupational Rehabilitation, Journal of Affective Disorders, American Journal of Health Promotion, Social Science and Medicine, International Journal of Occupational Safety and Ergonomics, International Journal of Workplace Health Management, Journal of Workplace Behavioral Health, and Journal of Nursing Management. Each journal offers unique perspectives and insights into presenteeism, whether by exploring its psychological determinants, impact on employee well-being, or organizational implications. Through their collective contributions, these sources advance presenteeism research, providing valuable knowledge for academics and practitioners striving to address this prevalent issue in the workplace.

In presenteeism research, several less prominent sources are making substantial contributions to understanding the complexities of this phenomenon. Journals such as the Journal of Occupational Medicine and Toxicology, the European Journal of Work and Organizational Psychology, and the Journal of Human Resource Management Review may have fewer documents than leading publications. Still, their significant citation counts indicate their influence in the field. These sources delve into various aspects of occupational health, organizational psychology, and human resource management, offering valuable insights into the drivers and consequences of presenteeism in the workplace.

Journals such as the International Journal of Occupational Safety and Ergonomics, Journal of Nursing Management, and Journal of Occupational and Organizational Psychology provide specialized perspectives on safety, nursing management, and organizational behavior, contributing to a holistic understanding of presenteeism’s impact on employee well-being. Despite their lower document counts, these sources attract attention from researchers and practitioners alike, reflecting their relevance and importance in addressing presenteeism-related challenges. While not as widely recognized as top-tier journals, these lesser-known sources play a crucial role in advancing presenteeism research and shaping strategies for promoting healthier and more productive work environments.

#### 4.1.2 Science mapping analysis

In bibliometrics, content analysis offers a systematic and objective way to research scholarly literature, allowing scholars to analyze vast quantities of publications and derive relevant conclusions about knowledge on a certain topic. It provides vital insights into research trends, influence, and impact, allowing researchers, institutions, and governments to make educated decisions and plan for future research endeavors.

##### 4.1.2.1 Co-occurrence network

Co-occurrence analysis with author keywords allows us to investigate the relationships and connections between the many terms authors use to describe their study. This analysis can uncover thematic clusters and highlight a discipline’s primary study topics and areas of interest. Researchers can acquire insights into the important themes and subjects being investigated by the scholarly community by recognizing the keywords that regularly co-occur together. Furthermore, co-occurrence analysis with author keywords might aid in identifying developing trends or study areas of interest. Researchers can locate fresh and evolving study subjects by detecting increasing frequency and co-occurring keywords with other related terms. This data can be useful for keeping up with the newest advances and identifying potential research topics.

*Cluster 1: Social Interaction and Emotional Stress.*

Social interaction and emotional stress have emerged as significant factors influencing employees’ ability to perform optimally and contributing to presenteeism, a condition in which people are physically present but are not fully engaged in their work. Engaging with others in various social contexts is referred to as social interaction. Several studies have found a link between social interaction and decreased presenteeism. Positive workplace social interactions can foster a sense of belonging, social support, and a collaborative work environment, ultimately reducing presenteeism. Employees’ well-being and productivity can suffer as a result of emotional stress, which manifests as feelings of anxiety, exhaustion, and burnout. Emotional stress and presenteeism have consistently been linked in studies.

Stress can impair cognitive functioning, decision-making skills, and job performance. Reducing emotional stress at work is critical for reducing absenteeism and improving overall employee well-being. Employee engagement, satisfaction, and overall performance will likely improve in organizations prioritising social interaction and implementing interventions to reduce emotional stress. More research is required to investigate the mechanisms underlying these relationships and develop targeted interventions to reduce presenteeism. High amounts of stress can affect cognitive functioning, decision-making abilities, and job effectiveness. Reducing emotional stress in the workplace is vital for lowering presenteeism and improving overall employee well-being.

*Cluster 2: Psychological Aspects of Health.*

Cluster 2 keywords delve into the psychological elements that contribute to presenteeism. It investigates how stress, including work-related pressures, affects employees’ capacity to focus and perform efficiently. It also explores the impact of presenteeism on job satisfaction, engagement, motivation, and burnout. This demonstrates the links between these psychological characteristics and employees’ presence and performance at work. Presenteeism has an impact not just on work productivity but also on employees’ overall health and well-being. To effectively treat presenteeism, organizations must develop intervention measures focusing on psychological health elements. These keywords cover many techniques: stress management programs, employee well-being initiatives, flexible work arrangements, and supportive leadership practices. The cluster also implies the possible benefits of these strategies in reducing presenteeism and boosting psychological well-being among employees. Organizations prioritizing employee psychological well-being and adopting focused treatments are more likely to minimize presenteeism and promote a better workplace.

*Cluster 3: Employee wellbeing and productivity.*

It emphasizes the significance of productivity as a crucial consequence of well-being and explores presenteeism’s negative implications on worker well-being and organizational performance. This cluster lays the groundwork for comprehending the relationship between employee well-being, productivity, and presenteeism. It investigates the effects of physical, psychological, and social well-being elements on employee productivity, such as work-life balance, job satisfaction, engagement, and supportive working environments. The section examines empirical evidence emphasizing the favorable relationships between well-being and work efficiency. It underlines that promoting employee well-being is morally important, increases productivity, and decreases presenteeism, which benefits individuals and organizations. Organizations can effectively manage presenteeism and develop a culture of productivity and flourishing by employing initiatives that improve employee well-being and establish a supportive work environment.

*Cluster 4: Employee Health Outcome.*

It investigates how different health aspects, such as physical and mental health and chronic illnesses, influence the occurrence and effects of presenteeism. It examines how chronic diseases, discomfort, weariness, and overall physical well-being contribute to presenteeism. This cluster includes empirical data associating physical health problems with lower productivity and a higher probability of presenteeism. Their mental health greatly influences employee well-being and productivity. This section investigates the link between mental health disorders such as anxiety, depression, and stress and presenteeism. It examines how poor mental health might contribute to presenteeism by impairing cognitive performance, decision-making, and involvement. Presenteeism has an impact not only on productivity but also on employee health outcomes. It emphasizes the importance of addressing presenteeism to mitigate these poor health impacts.

*Cluster 5: Organization Culture and Support.*

**Figure 3:**
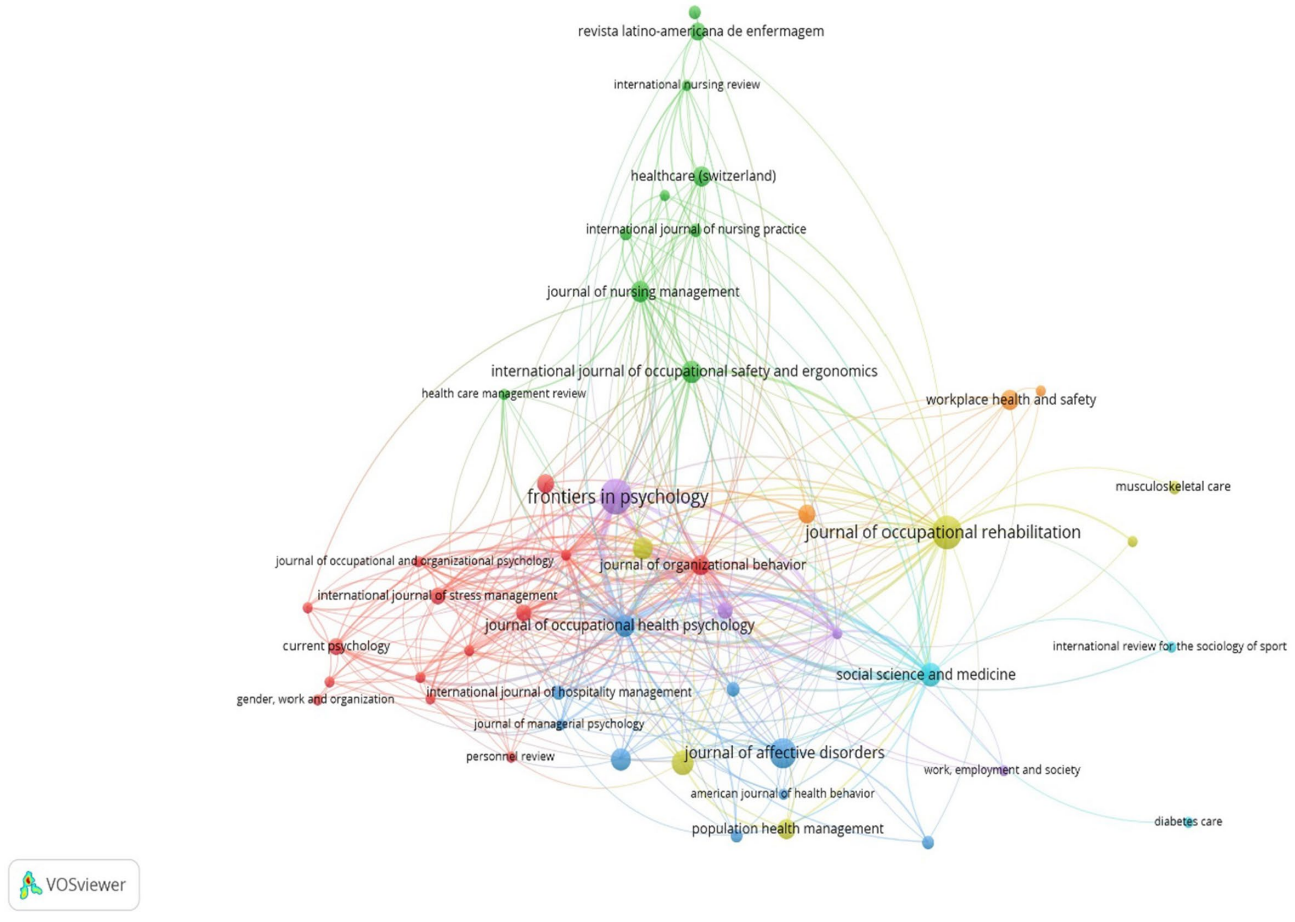
Citation analysis on sources of presenteeism research.

It investigates how elements of organizational cultures, such as leadership styles, norms, values, and support structures, influence the occurrence and effects of employee presenteeism. Understanding these factors is critical for organizations seeking to establish a culture that reduces presenteeism while fostering a healthy and productive staff. It focuses on the role of organizational factors in creating employee behaviors and attitudes toward presenteeism. Leadership is critical in influencing employee behaviors and building organizational culture. A positive work atmosphere is essential for reducing absenteeism and fostering employee well-being. It investigates the effect of social support from coworkers and supervisors in minimizing presenteeism and promoting a supportive and collaborative atmosphere. To effectively handle presenteeism, open communication and understanding are required. It explores how awareness campaigns, training programs, and educational initiatives can help reduce stigma, increase knowledge, and encourage employees to prioritize their health above presenteeism (Figure 3).

*Cluster 6: Work-life integration.*

**Figure 4:**
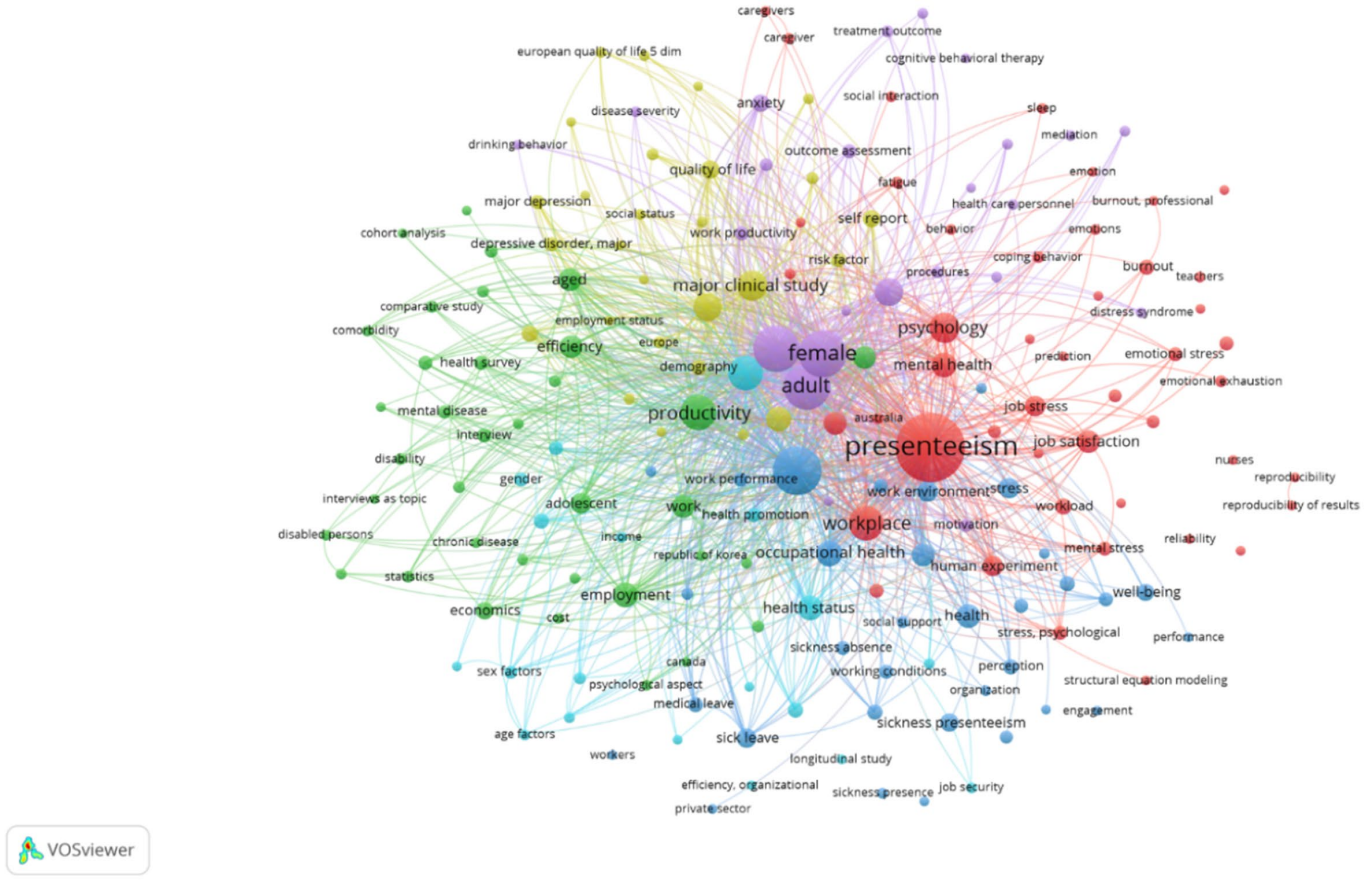
Co-occurrence network.

It investigates how the balancing of work and home life affects the occurrence and effects of employee presenteeism. Understanding the dynamics of work-life integration and presenteeism is critical for organizations seeking to foster a peaceful and healthy work environment. It investigates how the increasing integration of work into personal life through technology and flexible work arrangements can outline boundaries and make disengagement from work harder. The section addresses how work-life integration might benefit from constant connectivity and high job expectations. Balancing work and personal life can lead to greater stress, a loss of work-life balance, and an increased risk of burnout. It investigates the effect of poor well-being on presenteeism and the potential ramifications for physical and mental health outcomes. It investigates how high job expectations, lengthy working hours, and an inability to unplug from work might harm employee performance, efficiency, and creativity (Figure 4).

##### 4.1.2.2 Bibliographic coupling

**Figure 5:**
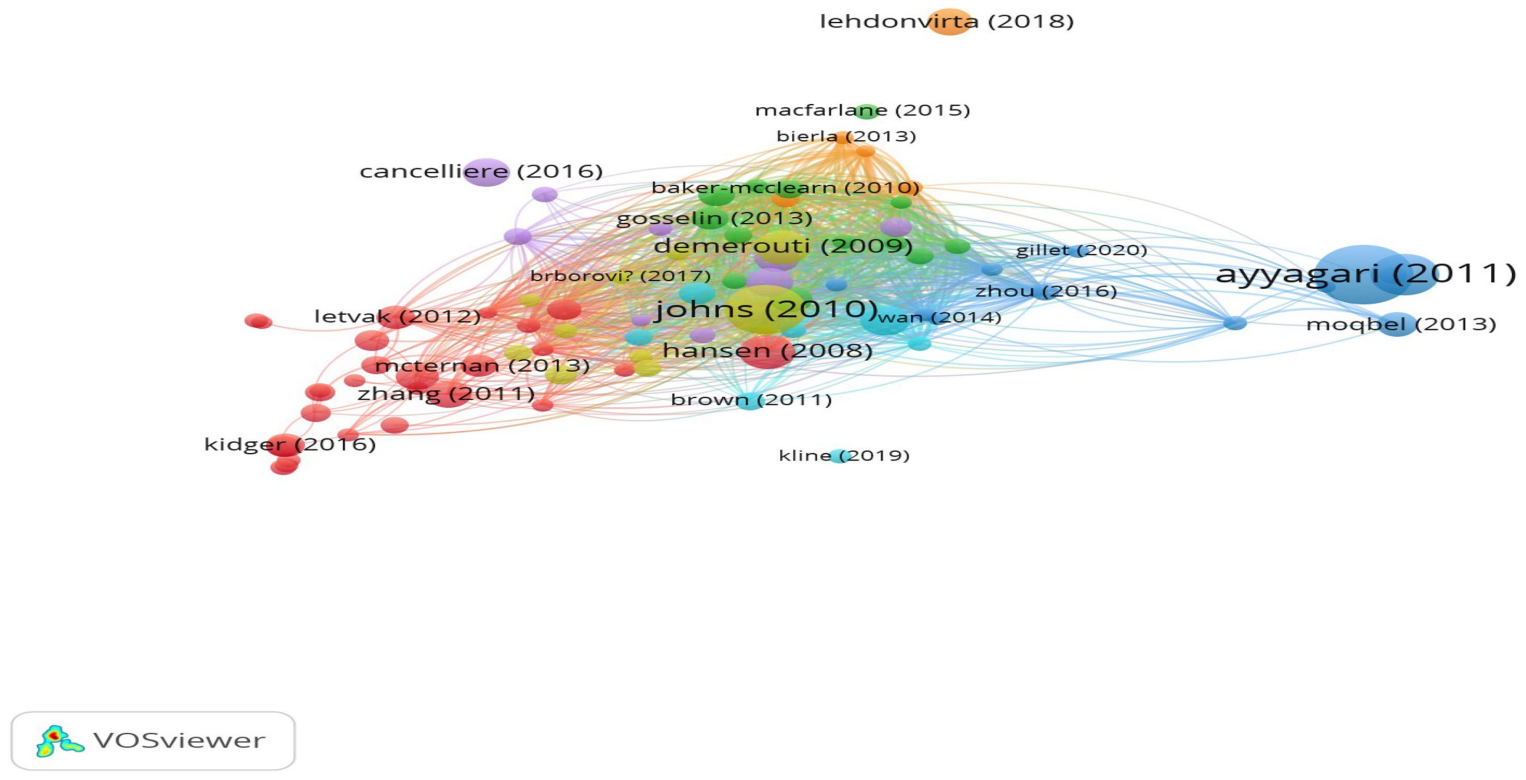
Bibliographic coupling.

Figure 5 Represents the Bibliographic coupling map of Presenteeism research. The bibliographic coupling map comprises 88 articles based on total bibliographic coupling link strength. As highlighted by the bibliographic coupling map in different colors, these 88 articles have been grouped into eight clusters consisting of 27 articles in Cluster 1, 13 articles in Cluster 2, 12 articles in Cluster 3, 11 articles in Cluster 4, 9 articles in cluster 5, 7 articles in cluster 6, 5 articles in cluster 7, and 4 articles in cluster8. The articles belonging to eight different clusters are further utilized to extract the intellectual structure of presenteeism research. The content analysis section has analyzed and discussed all the relevant articles of the bibliographic coupling map.

*RQ3*: What are the key concepts that have been explored on the topic of presenteeism, and how are they related.

##### 4.1.2.3 Co-citation network

**Figure 6:**
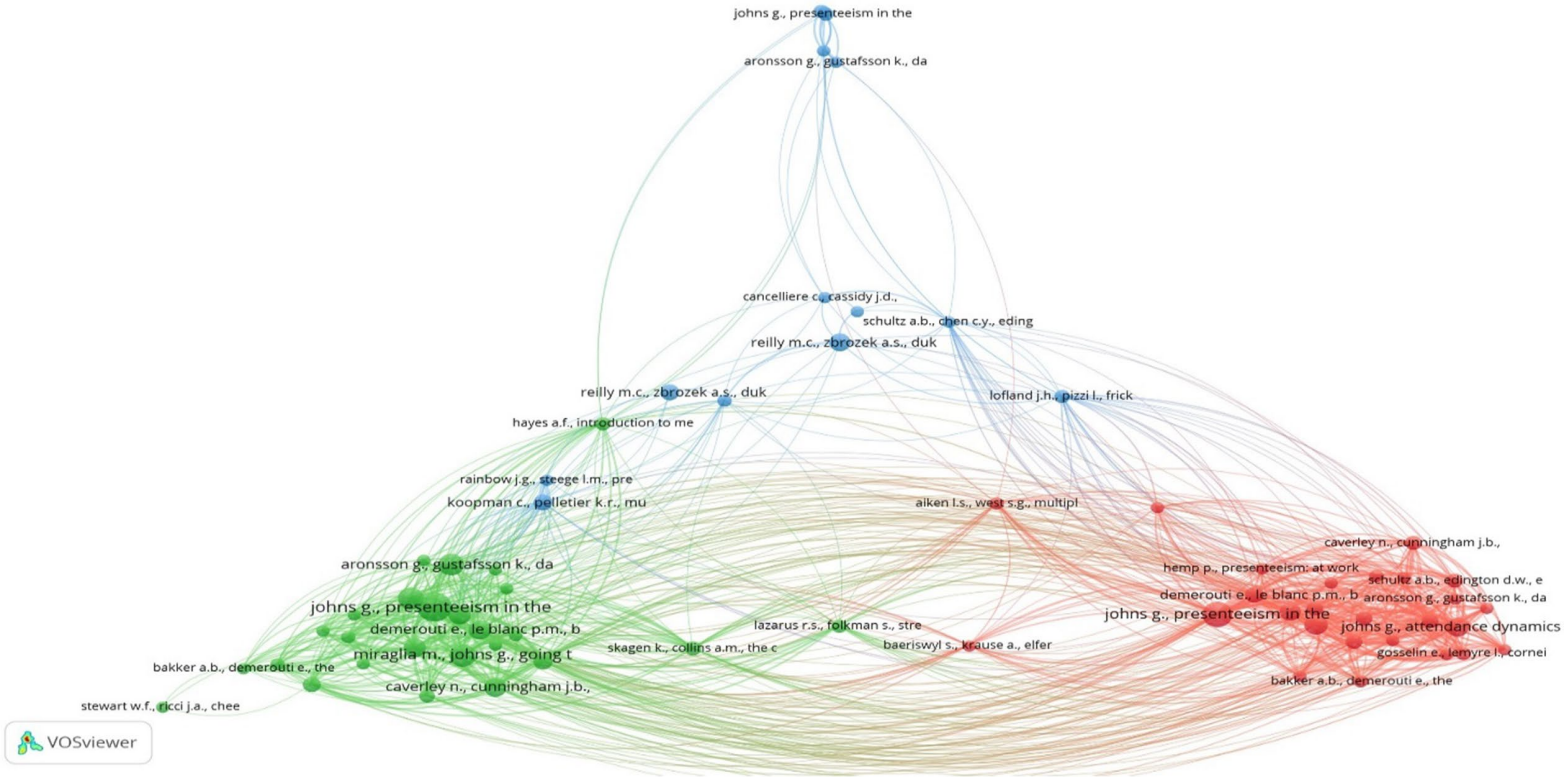
Co-citation network.

Figure 6 reveals the co-citation map of the ERA of Presenteeism research. The co-citation map consists of the top 66 articles based on the co-cited articles’ total co-citation link strength. As highlighted by the co-citation map in different colors, these 66 articles have been grouped into three clusters consisting of 26 articles in Cluster 1, 26 in Cluster 2, and 14 in Cluster 3. The articles belonging to three different clusters are further utilized to extract the intellectual structure of presenteeism research. All the relevant articles of the co-citation map have been analyzed and discussed in the content analysis section.

##### 4.1.2.4 Country collaboration

*RQ4:* What is the nature of cooperation that is evident in the publication of presenteeism.

**Figure 7:**
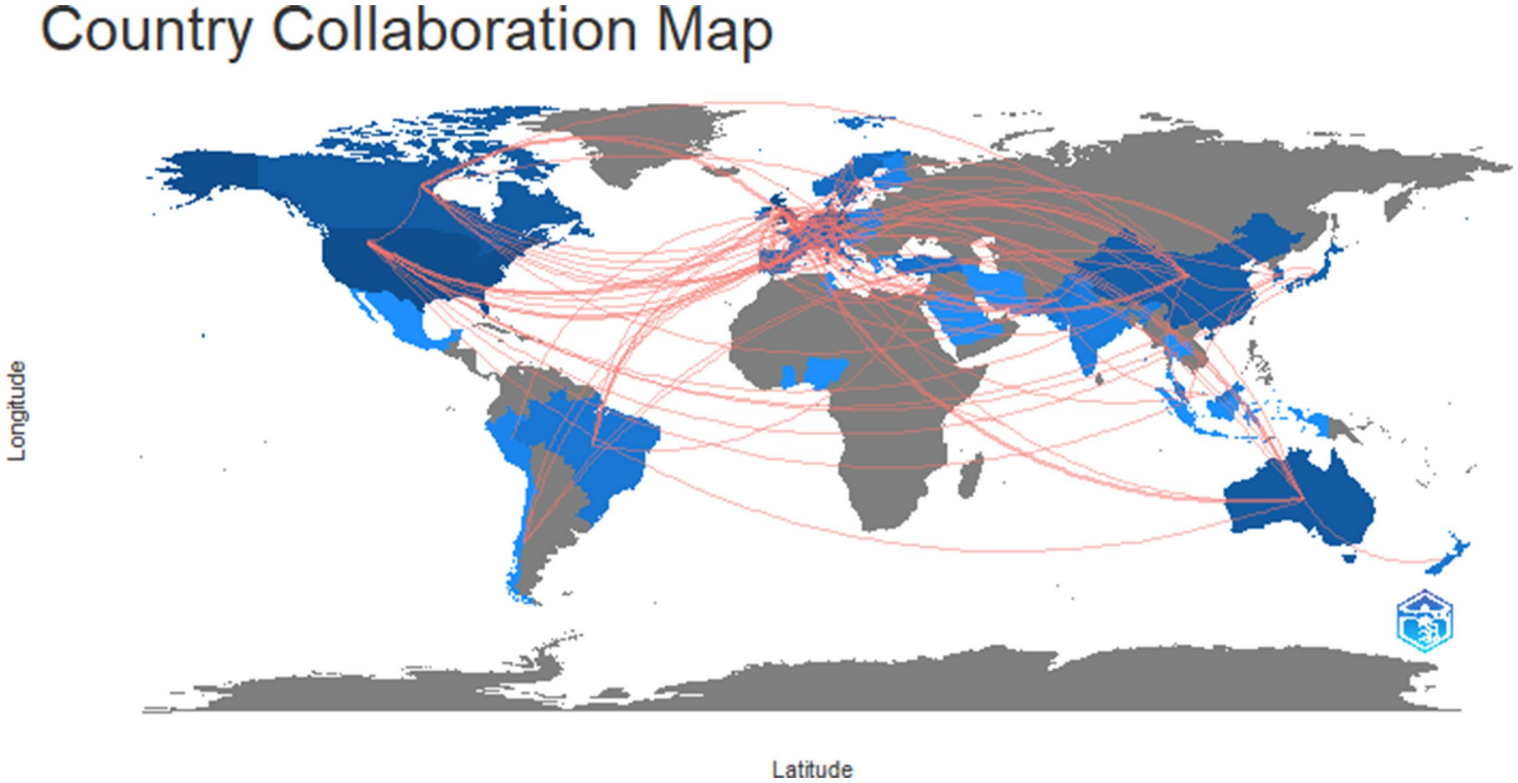
Country collaboration.

Figure 7 provides a thorough interpretation of the results of the bibliometric study performed with R Studio. It delves into the significance of the found collaborative patterns, exploring the elements that lead to effective country collaboration and the possible benefits of such collaborations. The figure shows that most of the countries’ authors collaborate on the research area of presenteeism. Further, we can say that most of the studies are collaborative work.

##### 4.1.2.5 Thematic map

**Figure 8:**
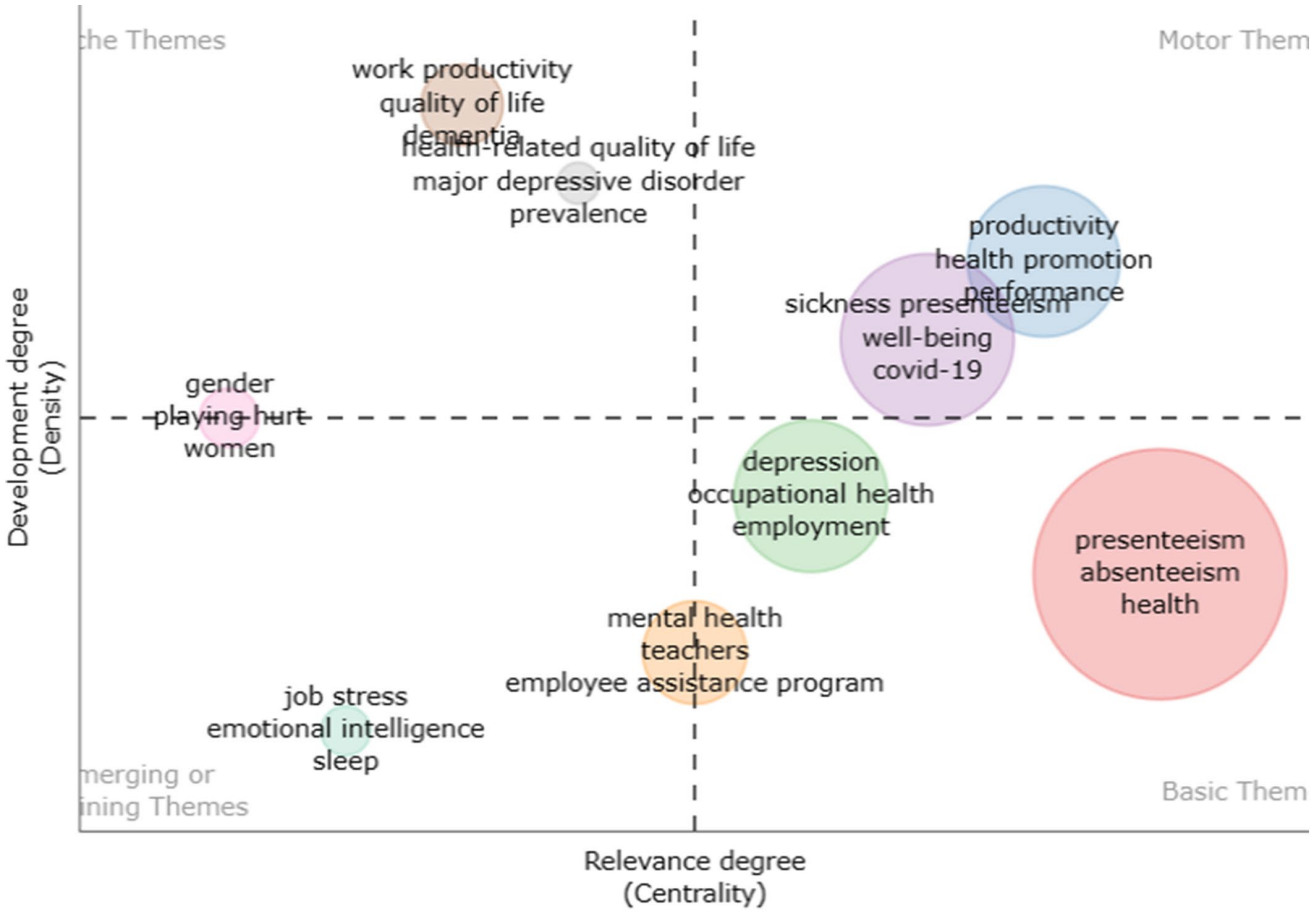
Thematic map.

Figure 8 represents a thematic map using all keywords. The figure is divided into four themes: basic theme, motor theme, niche theme, and emerging theme, each characterized by centrality and density. The basic theme consists of keywords such as presenteeism, absenteeism, and health, which have been extensively studied. Numerous studies on productivity, health promotion, performance, COVID-19, and well-being have indicated a focus on motor studies. The niche theme encompasses quality of life and major depressive disorder. The emerging themes in presenteeism include job stress, emotional intelligence, and sleep. These themes require further exploration based on gender, mental health, and employee assistance programs.

### 4.2 Content analysis

*RQ5:* What is the intellectual structure of knowledge-base on presenteeism.

Based on the content analysis conducted on abstracts of 154 key research articles, the intellectual structure of presenteeism research emerged with five themes.

Theme 1: Workplace Presenteeism and Employee Health.

Theme 2: Medical Conditions and Health Costs.

Theme 3: Work Productivity and Well-being.

Theme 4: Testing and Measurement.

Theme 5: Miscellaneous Factors.

Theme 1: Workplace Presenteeism and Employee Health.

This Theme revolves around the concept of presenteeism in the workplace, which refers to employees being physically present but not fully productive due to health issues. It explores attendance pressure, sickness presenteeism, and work-related factors that affect employee health and well-being. The context also considers the impact of presenteeism on general health, public health hazards, and nurses’ perceptions regarding presenteeism. The studies that come under this context are Workplace presenteeism, Attendance Pressure Factors, Sickness Presenteeism, Work-related factors, Impact of attendance, General health, Public health hazard, Nurses Presenteeism, Consequences on health and wellbeing, Nurse perceptions on presenteeism and Positive and negative effects.

Theme 2: Medical Conditions and Health Costs.

This Theme focuses on the relationship between medical conditions and the costs associated with presenteeism. It includes the influence of medical conditions, such as depression, on presenteeism and the health and financial implications for employers. The context also considers recent trends in presenteeism and the costs incurred due to presenteeism. It contains the context of Medical Conditions, Depression, Employers’ health and cost due to presenteeism, Nurse”s Presenteeism, Recent trends in presenteeism, and Presenteeism costs.

Theme 3: Work Productivity and Well-being.

In this theme, the emphasis is on the relationship between work productivity and employee well-being. It explores how employee health status impacts their productivity and overall organizational performance. The context also considers presenteeism’s economic perspective, leaders’ role in presenteeism, and the importance of a psychosocial safety climate for employee well-being. Additionally, it includes potential research directions in this field. It includes Health status and employee productivity, Work-related factors, Well-being, Economic Perspective of presenteeism, Leader Presenteeism, and Psychosocial safety climate.

Theme 4: Testing and Measurement.

This Theme centres around testing and measurement related to presenteeism. It involves developing and using scales or tests to assess presenteeism, potentially across different cultures. The context also considers the presence of nurse presenteeism and identifies potential research directions for further exploration. The Themes included are Test for scale, Cross-cultural study, Nurse Presenteeism, and Research Directions.

*Theme 5: Miscellaneous Factors.*

This Theme includes various factors that are not directly tied to a specific theme but are still relevant to presenteeism. It encompasses the involvement of physicians in addressing presenteeism, the impact of cyberbullying and virtual presenteeism, the influence of the COVID-19 pandemic on presenteeism, and the relationship between psychological distress and presenteeism. Additionally, it explores the effects of sleep duration, quality, and rhythm on presenteeism. It includes Physicians, Cyberbullying, Virtual presenteeism.

Table 7 presents a comprehensive overview of presenteeism research across various organizational contexts, highlighting the diverse motives, positive consequences, and negative consequences associated with employees’ decisions to work while ill or impaired. In healthcare organizations, the sense of duty to patients emerges as a primary motivator, leading to positive outcomes such as the continuity of patient care. However, this medication can also compromise patient safety due to illness transmission from healthcare workers. Similarly, in corporate settings, fear of job loss and pressure to meet deadlines often drive presenteeism, contributing to perceived dedication to the job and leading to negative consequences such as reduced productivity and increased burnout.

**Table 7:** Motives and consequences of presenteeism in the organizational context.

Organizational Context	Motives of Presenteeism	Positive Consequences	Negative Consequences
Healthcare organizations (Al Nuhait et al., 2017; Lui et al., 2018; Kuster et al., 2021; Lichtman et al., 2021; Nogueira and De Oliveira, 2023)	Sense of duty to patients	Continuity of patient care	Compromised patient safety
	Fear of burdening colleagues	Reduced workload for co-workers	Transmission of illness to patients
	Concerns about job security	Perceived dedication to the profession	Personal health deterioration
Corporate organizations (Musich et al., 2006; D’Abate and Eddy, 2007; Schultz et al., 2009; Wang et al., 2023)	Fear of job loss	Perceived dedication to the job	Reduced productivity
	Desire to meet deadlines	Reduced absenteeism	Burnout and stress
	Pressure from supervisors	Improved team morale	Detrimental effects on work quality
Educational institutions (Ferreira and Martinez, 2012; Kinman and Wray, 2018; Hadjisolomou et al., 2022; Uslukaya et al., 2022)	Desire to meet academic expectations	Academic achievement	Decreased student engagement
	Fear of falling behind peers	Recognition from faculty	Increased stress and anxiety
	Limited availability of substitutes	Sense of accomplishment	Negative impact on learning outcomes
Government agencies (Irvine, 2011; Taifor and Abdullah, 2011)	Sense of duty to the public	Continued public service delivery	Decreased efficiency in public services
	Political pressure	Recognition for dedication to duty	Public safety risks
	Career advancement aspirations	Meeting legislative requirements	Government accountability concerns
Nonprofit organizations (Markussen et al., 2010; Huff and Ablah, 2016; Gross et al., 2019; Maurício and Laranjeira, 2023)	Commitment to the cause	Fulfillment of organizational mission	Increased workload for others
	Desire to make a difference	Sense of accomplishment	Impact on service quality
	Limited resources for replacements	Improved organizational resilience	Burnout and turnover
Small businesses (D’Abate and Eddy, 2007; Schwatka et al., 2018; Knani et al., 2021)	Fear of financial repercussions	Business continuity	Long-term negative health effects
	Concerns about client relationships	Enhanced reputation	Reduced job satisfaction
	Limited staffing resources	Increased revenue	Decreased work-life balance
Manufacturing companies (Dew et al., 2005; Fernando et al., 2017; Mori et al., 2022)	Pressure to meet production targets	Continuity of production	Safety hazards and accidents
	Concerns about job security	Reduced downtime	Increased absenteeism
	Economic incentives	Enhanced competitiveness	Occupational injuries
Retail Chains (Palmer et al., 2010; Fernando et al., 2017)	Desire to meet sales targets	Maintained customer satisfaction	Decreased employee morale
	Fear of repercussions from management	Improved sales performance	Higher turnover rates
	Limited staffing resources	Enhanced brand reputation	Decreased work-life balance

Educational institutions face unique challenges with presenteeism, where the desire to meet academic expectations and fear of falling behind peers motivate students and faculty to work while ill. While this may result in educational achievement and recognition, it can also decrease student engagement and heightened stress levels. Government agencies grapple with presenteeism driven by a sense of duty to the public and political pressure, which can maintain public service delivery but decrease efficiency and pose public safety risks. Nonprofit organizations experience presenteeism stemming from commitment to the cause and desire to make a difference, leading to increased workload for others and potential burnout.

In small businesses, concerns about financial repercussions and client relationships motivate presenteeism, resulting in business continuity, enhanced reputation, and long-term negative health effects and decreased job satisfaction. Manufacturing companies face pressure to meet production targets and concerns about job security, driving presenteeism that ensures continuity of production but also leads to safety hazards and increased absenteeism. Finally, in retail chains, the desire to meet sales targets and fear of repercussions from management motivate presenteeism, contributing to maintained customer satisfaction and decreased employee morale and work-life balance issues. Overall, the table underscores the complexity of presenteeism across diverse organizational contexts, highlighting its benefits and drawbacks.

### 4.3 Theory, characteristics, context, and methodology framework and future research avenues

Table 8 represents the TCCM framework on Presenteeism research. This study draws on various theoretical frameworks to understand the underlying motivations and consequences of presenteeism behavior. The theories mentioned in the table, such as Social Exchange Theory, Conservation of Resource Theory, Job Demands-Resources Model, and Effort-Reward Imbalance Model, offer valuable perspectives on how individuals weigh the costs and benefits of attending work while unwell or facing other challenges. These theories help researchers explore factors like reciprocity, resource depletion, job demands and resources, and the workplace’s balance between effort and reward.

**Table 8:** TCCM framework.

Theory	Authors
Social exchange theory	Wu et al. (2023)
Conservation of resource theory	Choi et al. (2024)
Job demands-resources model	Vinod Nair et al. (2020)
Effort-reward imbalance model	Smektala et al. (2019)
Job demand-control model	Jourdain and Vézina (2014)
Stressor-strain model	Chen et al. (2021)
Health belief model	Lohaus et al. (2021)
Transactional model of stress and coping	Nath et al. (2024)
Theory of planned behavior	Dietz et al. (2020)
Health action process approach (HAPA)	Rollo and Prapavessis (2021)
Biopsychosocial model	Frutiger et al. (2019)
Organizational support theory	Côté et al. (2021)
Job embeddedness theory	Liu et al. (2022)
Self-determination theory	Coutu et al. (2015)
Social cognitive theory	Cooper and Lu (2016)
**Characteristics**	
***Antecedents***	
High workload	Wang et al. (2018)
Job insecurity	Kim et al. (2020)
Poor work-life balance	Hwang and Jung (2021)
Organizational culture	Chang et al. (2015); Lui et al. (2024)
job autonomy	Mach et al. (2018)
Job dissatisfaction	Burton et al. (2006)
Health problems (physical or mental)	Merrill et al. (2012)
Economic instability	Galon et al. (2014)
Insufficient social support	Lu et al. (2013)
Peer pressure	Daniels et al. (2021)
Inflexible workplace policies	Munir et al. (2008)
Personal Characteristics	Mandiracioglu et al. (2015)
***Outcomes***	
Reduced productivity	Johns (2011)
Quality decline	Itani et al. (2022)
Error likelihood	Niven and Ciborowska (2015)
Job dissatisfaction	Karanika-Murray et al. (2015)
Morale decline	Pärli (2018)
Healthcare costs	Schultz et al. (2009)
Employee burnout	Demerouti et al. (2009)
Increased stress	Van Der Feltz-Cornelis et al. (2020)
**Context**	
Public service	Gosselin et al. (2013)
Healthcare	Challener et al., (2021); Yang et al., (2017); Lichtman et al., (2021); Yildiz et al., (2017)
Social work sectors	Janssens et al. (2013)
Manufacturing company	Nowak et al. (2023)
Banking sector	Sarwat et al. (2021)
Tourism industry	Arslaner and Boylu (2017)
Hospitality industry	Knani (2022)
Telecom sector	Jung and Jung (2015)
Education sector	Scuffham et al. (2014)
**Methodology**	
Qualitative study: meta-analysis	Miraglia and Johns (2016)
Quantitative; cross-sectional	Janssens et al. (2015) Pohling et al. (2016)
Quantitative; longitudinal study	Dietz et al. (2020) and Poethke et al. (2023)
Qualitative; proof of concept study	Biron et al. (2022)
Quantitative, sectional, online survey	Van Der Feltz-Cornelis et al. (2020)
Qualitative, integrative study	Freeling et al. (2020)
Qualitative; systematic review	Woodland et al. (2023)
Quantitative: a multicenter cross-sectional study	Li et al. (2022)

The characteristics of presenteeism encompass both antecedents (factors that influence its occurrence) and consequences. Factors such as high workload, job insecurity, poor work-life balance, organizational culture, job autonomy, health problems, economic instability, and insufficient social support contribute to the prevalence of presenteeism. Understanding these characteristics allows researchers to identify the triggers and outcomes of presenteeism behavior, enabling interventions and policy recommendations to mitigate its negative impact on individuals and organizations.

Presenteeism occurs within a specific organizational, sectoral, and cultural context. The table highlights various industries and sectors where presenteeism is prevalent, including public service, healthcare, manufacturing, banking, tourism, hospitality, and education. Each context may have unique stressors, norms, and organizational structures that shape employees’ decisions to attend work despite being unwell. Examining presenteeism within different contexts provides insights into industry-specific challenges and informs targeted interventions to address them effectively.

Presenteeism research employs diverse methodologies to investigate its prevalence, determinants, and consequences. These include qualitative and quantitative approaches such as meta-analyses, cross-sectional studies, longitudinal studies, proof of concept studies, online surveys, integrative studies, systematic reviews, and multicentre studies. By utilizing multiple methodologies, researchers can triangulate findings, validate results, and gain a comprehensive understanding of presenteeism across different populations and settings. Additionally, rigorous methodological approaches enhance the reliability and validity of research findings, contributing to evidence-based interventions and policy recommendations.

#### 4.3.1 Future research avenues for presenteeism research based on the TCCM framework

- Theory Integration: Exploring how different theoretical frameworks intersect and complement each other in understanding presenteeism behavior.
- Longitudinal Studies: Conducting more longitudinal studies to track the development of presenteeism over time and identify long-term consequences.
- Contextual Analysis: Investigating how cultural, organizational, and industry-specific factors influence presenteeism behavior.
- Intervention Studies: Designing and evaluating interventions to mitigate the negative effects of presenteeism on individuals and organizations.
- Qualitative Exploration: Conducting in-depth qualitative studies to understand the subjective experiences and motivations behind presenteeism.
- Global Comparative Studies: Comparing presenteeism behavior across countries and regions to identify cultural differences and similarities.
- Exploring Context-Specific Interventions: Future research could focus on developing and evaluating interventions tailored to each organizational context’s unique characteristics and challenges. For example, in healthcare organizations, interventions might include flexible scheduling options or enhanced infection control measures to address illness transmission to patients.
- Understanding the Role of Organizational Culture: Further investigation is needed to know how culture influences presenteeism behavior and its consequences. Research could explore how norms, values, and leadership styles within different organizational contexts shape employees’ decisions to work while ill and the resulting outcomes.
- Examining the Impact of Technology: With the increasing reliance on technology-enabled remote work, future research could explore how technological advancements influence presenteeism across various organizational settings. This could involve investigating the use of telemedicine in healthcare organizations or the impact of remote work policies on presenteeism in corporate settings.

## 5 Discussion

This study aims to map out the landscape of presenteeism research in the workplace between 2000-and 2023 through bibliometric analysis and content analysis. The researchers have used Bibliometric analysis, which reveals the volume of research dedicated to understanding the impact of presenteeism on health conditions, productivity and well-being. Bibliometric analysis of presenteeism research also reveals several important insights with implications for theory and practice in organizational psychology. Firstly, the increasing trend in publications on presenteeism emphasizes its growing significance as a research topic, reflecting a recognition of its impact on organizational performance and employee well-being (Lohaus and Habermann, 2019; Schmidt et al., 2019). The publication trend of presenteeism research has seen significant growth, particularly since the year 2000, indicating increasing interest and recognition of the importance of this topic in both academic and practical contexts (Dietz et al., 2020). The cumulative total citations till 2023 were 11,498, reflecting the interest, impact, and influence of presenteeism research within the scholarly community. This analysis also highlights key contributors to presenteeism research, including authors, institutions, and countries. Prolific authors like Johns, G., Schaufeli, W. B., and Ferreira, A. have made significant contributions to presenteeism research. The researchers found that countries like the United Kingdom, the United States, and Canada lead in terms of citations, indicating their prominence in advancing knowledge on presenteeism. This finding is aligned with the result of (Karanika-Murray and Biron, 2020).

Additionally, Journals such as Frontiers in Psychology and the Journal of Occupational Health Psychology play a crucial role in disseminating research on this Presenteeism research. The co-occurrence network and bibliographic coupling maps of this study reveal thematic clusters and relationships between concepts within presenteeism research. Key themes such as social interaction, psychological aspects of health, employee well-being and productivity, and organizational culture emerge, providing insights into the multidimensional nature of presenteeism and its impact on various aspects of work and health.

The content analysis of this study identifies the following five main themes in presenteeism research: workplace presenteeism and employee health; medical conditions and health costs; work productivity and well-being; testing and measurement; and miscellaneous factors. The TCCM framework offers a theoretical foundation for understanding presenteeism behavior, its characteristics, contextual factors, and methodological approaches used in research. The framework draws on theories like Social Exchange Theory and Conservation of Resource Theory to explore the motivations and consequences of presenteeism, highlighting the complex interplay between individual, organizational, and environmental factors. The findings suggest organizations should adopt a holistic approach to addressing presenteeism, considering quantitative metrics such as publication trends and qualitative insights from content analysis. This approach can inform the design of interventions to reduce presenteeism and promote employee well-being. For instance, organizations may use bibliometric analysis to identify key researchers and journals in the presenteeism field. Moreover, content analysis can provide insights into the themes and topics that are more relevant to their employees. By integrating insights from both types of analysis, organizations can develop targeted interventions that address the underlying causes of presenteeism and create a healthier and more productive work environment. The identified thematic clusters and key themes from the content analysis of this study provide a foundation for theory development in organizational psychology. By synthesizing existing knowledge and identifying gaps, researchers can refine existing theories or develop new frameworks, instruments, and theories to enhance the dynamics of presenteeism.

Even though many researchers have shown an interest in presenteeism research. Still, our study findings mainly focused on thematic clustering, theoretical frameworks, practical implications, regional and institutional differences, and methodological variation, which creates interest among readers and future researchers to study presenteeism. These differences could stem from factors such as variations in coding criteria, disciplinary perspectives, cultural differences, and methodological approaches. Despite these potential discrepancies, this study contributes valuable insights to the understanding of presenteeism and its implications for theory and practice in organizational psychology. By addressing these discrepancies and building upon existing knowledge, researchers can further refine theoretical frameworks, develop evidence-based interventions, and create policies aimed at reducing presenteeism in the workplace and promoting employee well-being in diverse organizational contexts.

The findings of this study carry significant implications for theory development in organizational psychology. Through the synthesis of existing knowledge and identification of thematic clusters, the study provides a robust foundation for refining current theories or formulating new frameworks to elucidate the complexities of presenteeism. Adopting the TCCM framework and integrating theories such as Social Exchange Theory, Conservation of Resource Theory, and the Job Demands-Resources Model discussed in the study will help researchers gain a more nuanced understanding of the motivations and consequences of presenteeism within organizational contexts. Additionally, the identified thematic clusters, encompassing workplace presenteeism and employee health, medical conditions and health cost, work productivity and well-being, testing and measurement, and miscellaneous factors, offer valuable insights for the theory development of future researchers.

Presenteeism research contributes to our understanding of organizational behavior by exploring the factors influencing employee decision-making and behavior in the workplace. Future Researchers can identify drivers of presenteeism by investigating themes such as workplace health, productivity, well-being, and organizational culture and develop strategies to mitigate its negative impact. In this study, the researchers identified thematic clusters related to psychological aspects of health, employee well-being, and organizational culture, which highlights the interconnectedness of individual and organizational factors in shaping presenteeism behavior. Understanding these dynamics is essential for promoting a healthy and productive work environment. The findings offer insights that can inform interventions to reduce presenteeism and encourage employee well-being. By understanding the role of factors such as social interaction, emotional stress, and work-life integration, organizations can develop targeted interventions to address underlying causes of presenteeism. (Werapitiya and Opatha, 2015) have highlighted the significant impact of work-related anxieties on employees’ ability to perform effectively at work, often leading to presenteeism. The literature suggests three types of interventions to address this issue: physically-oriented, psychologically-oriented and organization-related. Physically-oriented interventions focus on promoting employees’ physical health through initiatives such as health promotion programs. Psychologically-oriented interventions aim to enhance employee well-being and productivity through activities like relaxation techniques or cognitive-behavioral psychotherapies. Organization-related interventions tackle presenteeism from an organizational perspective, encompassing strategies such as skill development, supervisor and coworker support, work flexibility, and workload management (Taylor et al., 2021). These interventions collectively target different aspects of the work environment to mitigate the impact of work-related anxieties and promote a healthier and more productive workforce.

**Figure 9:**
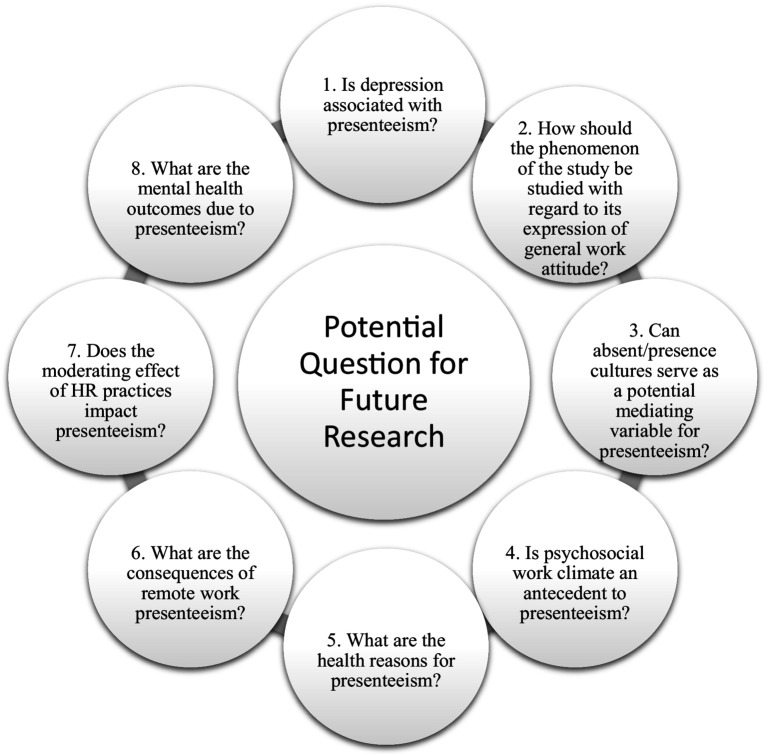
Research questions source: authors own creation based on the research data.

Interventions may include improving leadership support, fostering a positive work environment, promoting work-life balance, and providing resources for stress management and mental health support (Werapitiya and Opatha, 2015). Organizations can create conditions that support employee’s health and productivity by addressing these factors. Presenteeism research has implications for organizational policies and practices related to employee health and well-being (Cocker et al., 2011). By recognizing the impact of presenteeism on organizational performance and employee outcomes, policymakers and practitioners can develop policies and practices that prioritize employee health and create supportive work environments. For instance, organizations should understand the impact of presenteeism on employees’ productivity and performance (Mathieu and Gilbreath, 2023). They can implement flexible work arrangements, training on wellness programs, resilience, work-life balance, and policies encouraging employees to take time off when unwell. Organizations can reduce presenteeism and improve overall organizational performance by promoting a culture that values employee well-being. Figure 9 represents the potential question for future research.

## 6 Conclusion

The two-decade bibliometric analysis and content analysis of presenteeism trends revealed a significant increase in research output, reflecting a growing recognition of its impact on employee well-being and organizational productivity. The findings suggest a shift in focus from merely quantifying the phenomenon to exploring its underlying causes and implications. Foster a work environment that prioritizes employee well-being and encourages a healthy work-life balance. This study motivates organizations to take initiatives such as wellness programs, flexible work arrangements, and providing resources for stress management and mental health support to minimize presenteeism in the workplace.

It is suggested that the organization should ensure that employees feel comfortable taking sick leave when required without fear of repercussions. The organizations should communicate clearly about sick leave policies and procedures and emphasize the importance of staying home when unwell to prevent the spread of illness, promote faster recovery, and contribute more to the organization after recovering from illness. In certain circumferences, wherever it is feasible, the organizations should allow employees to work remotely or in hybrid mode when they are unwell or need to take care of a sick family member. Remote/ hybrid mode of work can reduce the need for presenteeism when the employees are ill, and the organization can maintain productivity. The organization should allow their employees to adjust their work hours or take breaks when it is required to accommodate their personal or family responsibilities, appointments, or health-related concerns. Flexibility in scheduling can reduce the pressure to attend work when unwell, which leads to lesser productivity and performance at the workplace. The organization should monitor employees’ workload and provide support or redistribute tasks as necessary to prevent burnout and excessive stress. The organization should encourage employees to open communication about workload concerns and provide resources for stress management and coping strategies. The organization should motivate their employees to prioritize self-care and maintain a healthy balance between work and personal life. This can involve setting boundaries for their employees concerning working hours, promoting time off for relaxation and recreation, and discouraging excessive overtime. The organization should provide training for managers and supervisors on recognizing signs of presenteeism creating a supportive work environment, and increasing awareness among employees about the negative effects of presenteeism on productivity, health, and overall well-being. Provide education on the importance of self-care, taking breaks, and seeking support when required. By implementing these strategies, organizations can create a healthier work environment that reduces the prevalence of presenteeism and supports the well-being and productivity of employees.

Finally, some recommendations for future research are presented. A deeper exploration of citation patterns could be undertaken to pinpoint influential works, emerging trends, and potential areas for further investigation within presenteeism. This could involve an in-depth analysis of citation networks and the identification of seminal papers, thereby providing researchers with more profound insights into the intellectual development in this field. Future researchers can find some more dimensions that have greater impact on presenteeism, build new theories on presenteeism and develop a new instrument for future survey-based research. Future research should focus on a longitudinal research design with a minimum of three waves to settle the causality should need to be studied. Longer-term longitudinal research is required to elucidate the influence of both macro-level economic and micro-level individual issues on presenteeism.

## Data availability statement

The raw data supporting the conclusions of this article will be made available by the authors, without undue reservation.

## Author contributions

DC: Methodology, Writing – original draft. VA: Writing – review & editing. AV: Supervision, Writing – review & editing.
